# 

**Published:** 1886-10-09

**Authors:** 


THE HOSPITALS ASSOCIATION,

NORFOLK HOUSE, NORFOLK STREET, STRAND, W.C.

*President*—SIR ANDREW CLARK, Bart., M.D., F.R.S.

EVERY INSTITUTION REPRESENTED BY THE MEMBERS OF THE ASSOCIATION, AND ALL ITS MEMBERS, ARE SUBSCRIBERS TO "THE HOSPITAL."

**NOTICE TO CORRESPONDENTS.**

*All correspondence must be written on one side of the paper only.*

*We cannot undertake to return MSS. not used.*

**Communications,** *whether intended for insertion or for private information, must be authenticated by the names and addresses of the writers, not necessarily for publication.*

**Local papers** *containing reports or news paragraphs* **should be marked.**

*It is especially requested that early intelligence of* **local events of interest,** *or which it may be thought desirable to bring under notice, may be sent direct to the Editor.*

*Communications respecting editorial matters should be addressed to the Editor, Norfolk House, Norfolk Street, Strand, London, W.C.*

**TERMS OF SUBSCRIPTION.**

*Subscribers to* THE HOSPITAL *residing in London and the Suburbs will receive their Copies by the first post on* FRIDAY MORNING. *Copies for Country Subscribers are forwarded by the* DAY *Mail on Fridays. Terms, including postage, payable in advance:*

| | Weekly Parts. | Monthly Parts. |
|---|---|---|
| Twelve months | 0 6 6 | 0 7 6 |
| Six " | 0 3 3 | 0 3 9 |
| Three " | 0 1 8 | 0 1 11 |

*Post Office Orders should be made payable at the General Post Office, London, E.C., to* WHITING AND Co., 30 *and* 32, *Sardinia Street, Lincoln's Inn Fields, W.C. Cheques to be crossed* "*London and Joint Stock Bank, Limited,*" *Chancery Lane Branch.*

**Cases for Binding** *the weekly or monthly parts of* THE HOSPITAL *in yearly vols. may be ordered of the Publishers.*

**BUSINESS COMMUNICATIONS AND ADVERTISEMENTS.**

*All communications concerning business matters, non-delivery of* THE HOSPITAL, *etc., should be addressed to the Manager, at the publishing offices,* 30 *and* 32, *Sardinia Street, Lincoln's Inn Fields, London, W.C.*

### The Hospital.

**An Institution, Family, and Parochial Journal of Hospitals, Asylums, and all Agencies for the Care of the Sick, Criticism and News.**

### WELL DONE!

### SPECIAL NOTICE.

A Gratifying Recognition.

Mr. W. Cuthbert Quilter, M.P., who has long taken a deep interest in the work of the hospitals, has sent us a cheque for £50, to be distributed in Prizes amongst the readers of The Hospital, at the discretion of Sir Andrew Clark, Bart., M.D., F.R.S., the President of the Hospitals Association, and the Editor. This is an act of practical sympathy in our work which we greatly appreciate, and the public-spirited generosity of Mr. Cuthbert Quilter cannot fail to be of substantial service to the hospitals in more ways than one. The conditions to be observed and the subjects selected for competition will be duly announced.

### "THE HOSPITAL" PRIZE COMPETITIONS.

We offer for competition among our readers money prizes, classified under the subjoined heads. We are anxious that this feature of The Hospital shall prove of interest to all classes of readers, and the grouping of the competitions has been arranged solely with this end in view.

All competitors who send in papers must strictly abide by the following

### Conditions.

I. Every paper sent in must be clearly written, and one side only must be used. All competitors must mark at the head of their papers the number of their competition.

II. Each paper must be signed by the author with his or her real name and address. A *nom de plume* may be added, if the writer does not desire to be referred to by us by his real name. In the case of all prize-winners, however, the real name and address will be published.

III. All communications relating to prize competitions should be addressed to "The Prize Editor, *The Hospital* Office, Norfolk House, Norfolk Street, Strand, W.C." Competitors are requested not to include in letters, etc., to the Prize Editor communications on matters of other business. All letters must be fully prepaid.

IV. The Prize Editor of *The Hospital* is the sole judge of the competitions, and his decision is in all cases final and without appeal.

**V. All competitions must be received at "The Hospital" Office by the dates stated, failing which they cannot be dealt with. Every Reply must be accompanied by the Prize Competition "Coupon" which appears on another page.**

VI. The Prize Editor reserves the right to publish any paper sent in, whether it receives a prize or not. He does not hold himself responsible for any MSS. sent, nor can he undertake to return rejected competitions.

VII. No competition must exceed two columns in length, except the Story competition, which must not exceed six columns.

No. 5.—The One Guinea Prize.

A prize of *One Guinea* will be offered every fortnight to all readers of *The Hospital* for the best *Story* or narrative based upon hospital, asylum, or student life, or upon any incident connected with the professions of medicine or nursing. The Prize Competition will be published in *The Hospital*.

Competitions must be received by Saturday, 16th October. Envelopes to be marked in left-hand corner, "Story."

No. 6.—The Half-Guinea Prize.

A prize of *Half-a-Guinea* is offered fortnightly to in and out-patients for the best *Anecdote* or *Personal Reminiscence* of any interesting circumstance which may have come under their observation while at or attending any hospital.

Competitions must be received by Saturday, 16th October. Envelopes to be marked in left-hand corner, "Anecdote" or "Reminiscence."

No. 7.—The Five Shilling Prize.

A Prize of *Five Shillings* is offered weekly to all readeis of *The Hospital* for the best *Item of News* suitable for publication in this Journal.

Competitions must be received by Saturday, 23rd October. Envelopes to be marked in left-hand corner, "News."

No. 8.—The Nine Pounds Quarterly Prizes.

Prizes of *Five, Three,* and *One Pound* will be awarded on 2nd January, 1887, to the three persons who shall have obtained during the months of October, November, and December, 1886, the largest number of subscribers to *The Hospital*. Order Forms, for subscribers to fill up and sign, may be obtained on application to the publishers, 30 and 32, Sardinia Street, Lincoln's Inn Fields, London, W.C. The Prize Editor must be informed by competitors from time to time of the names and addresses of new subscribers they procure. Names will be received up to and including Saturday, 23rd December, 1886.

### "THE HOSPITAL" CHARITY BOX.

Our "Charity Box" is intended to afford a little practical help to the hospitals. Each week we shall propose a word for "anatomical dissection," and the first 150 competitors who extract the largest number of words from it will receive the following prizes :—

| | | |
|---|---|---|
| 1st | ... ... | One Guinea. |
| 2nd | ... ... | Half a Guinea. |
| 3rd | ... ... | Five Shillings. |

The above Prizes will be repeated for every additional 150 competitors who may enter each week.

First Competition.

Compose the largest number of words you can from the name—

LINACRE.

*Observe:* Proper names, abbreviations, foreign words, and repetitions are barred.

Write only on one side of the paper, and total your words.

Append your real name and address, and add, if desired, a pseudonym for publication.

All replies, addressed "Charity Box," The Hospital, Norfolk House, Norfolk Street, W.C., must arrive by Saturday, October 16th. Results will appear Saturday, October 23rd.

*Every competitor is required to cut out and enclose with his list the "Charity Box" Coupon* (see advertisement pages), *and a shilling entrance fee.*

The proceeds resulting from each "Charity Box" competition will be funded until the end of December 1886. The total sum available for distribution, and the names of the hospitals selected for donations, will appear on Saturday, January 8, 1887. The distribution of the proceeds will be at the Editor's sole discretion.

### THE CHILDREN'S CORNER.

I refrain until next week from commenting on the response which my little friends are making. Then I shall have gathered the opinions of many into whose hands the first number of this Journal did not fall in time for their replies to receive notice in to-day's issue. I ought, perhaps, to have said last week that the "Children's Corner" is not intended for the children of an "older growth" than 16, as far as the competitions are concerned.

### OUR PASTIMES.

Children's Quarterly Prize Competition—

| | |
|---|---|
| Began ... ... ... | 2nd Oct. 1886 |
| Ends ... ... ... | 25th Dec. 1886 |

PRIZES.

| | |
|---|---|
| 1st ... ... ... | Thirty shillings. |
| 2nd ... ... ... | Twenty " |
| 3rd ... ... ... | Ten " |
| 4th ... ... ... | Five " |

will be awarded to the four competitors who shall have sent in, during the thirteen weeks, the largest number of correct answers to our pastimes.

5. I am the beginning of eternity,
The end of time and space,
The beginning of every end,
And the end of every place.
Who can guess this?

6. Transformed Words.—Change (*a*) *East* into *West*, and (*b*) *Skull* into *Greek*, altering only one letter at a time, making each time a legitimate English word. (As my young readers may not be acquainted with this amusing game, I will give an instance. Suppose we want to change *Heal* into *Many*. We proceed thus: *Heal* to *Many*—heal, heat, meat, melt, malt, male, mane, *many*.)

*East* to *West* and *Skull* to *Greek* must be worked out in a precisely similar way.

7. Who among my little readers can render into English the following Latin words?—

Is ab ille haeres ago
Fortibus es in aro.

8. Suppose you hailed a shoeblack from the other side of the street, the name of what London firm would you probably mention?

9. Tell me what two words possess all the five vowels in their alphabetical order?

Answers having the actual name and address (to which a pseudonym may be added for publication) must have "The Children's" Coupon attached (see advertisement pages), addressed to the "Puzzle Editor, The Hospital, Norfolk House, Norfolk Street, Strand, W.C.," not later than Thursday, 14th October. Results will appear in our issue of the 23rd inst.

### COLUMN FOR THE CURIOUS.

To be Sure.—Why are successful medical practitioners unlikely to be in a hurry? Because, don't you see, they have plenty of patients!

Dislike of Doctors.—Some people have a remarkable antipathy to doctors. An old man, who was dangerously sick, was urged by his friends to take the advice of a physician, but he persisted in his refusal, urging that he wished *to die a natural death.*

A cute young man went to the dentist's to have a tooth taken out. It was a troublesome job, and so the dentist advised him to take the gas. Before submitting himself to the influence of nitrous oxide, he dived his hand into his trousers-pocket. "Oh, never mind payment now," observed the dentist; "after the operation will do." "That's right enough," rejoined the cute young man, "but I intends to reckon up my cash before I take that gas of yours."

Brains.—There is a good story told of Dr. Hill, an Edinburgh professor of the last century. For many years, when he took his walks abroad in the streets, he used to be accosted by a half-witted individual who always had something foolish to say to him. At last the doctor grew so exasperated, that when next he met him, he said, "Well, Tom, you're still alive. Do you know how long one can live without brains?" "I dinna ken," said Tom. "How lang hae ye lived yersel'?"

Against the south wall on the outside of the old Church at Chelsea is a stone slab very quaintly inscribed in Latin, to the memory of Dr. Edward Chamberlayne, who died in the village in 1703. "Novem *liberos* genuit, sex *libros* composuit" (Nine children he had, six books he wrote), punningly writes his friend Walter Harris, doctor of physic. But the strangest part of the epitaph is lower down. So studious was Dr. Chamberlayne for the good of generations to come, that, continues Dr. Harris, he ordered a set of his books, covered with wax, to be buried with him, that they might be of use in time to come. "Dr. Harris evinced some singularity of opinion," says a local historian, commenting on the above, "in supposing that posterity would gain any information from works thus entombed with the body of their author. But whatever might have been the intention, his views in depositing the books in the tomb of his friend have been frustrated. Some years since, Dr. Chamberlayne's tomb yielded to the injuries of time, and, on examination, it was discovered that the damp and moisture admitted by the general decay had totally obliterated almost every appearance of the books; his seal, with his arms, was, however, still perfect."

Tight-lacing.—A doctor considers tight-lacing a public benefit, inasmuch as it kills off the foolish girls and leaves the wise ones to grow into women.

### THE OUT-PATIENTS' HOSPITAL DIARY.

*Continued from p.* 16.

The following Table gives the terms of admission, the names of the physicians and surgeons, and the days and hours of their attendance upon the out-patients at all the special hospitals in the Metropolis.

### CANCER.

| Hospitals and Terms of Admission. | Medical Officers, and Days and Hours of Attendance. Physicians. | Surgeons. |
|---|---|---|
| Cancer Hospital, Brompton, S.W. Entirely free | Dr. A. Marsden<br>„ H. Snow<br>„ F. A. Purcell<br>M., F., 2 | Mr. Jessett<br>„ Elam<br>„ C. Stonham<br>„ Jennings<br>Tu., W., Th., S., 2 |
| London Hospital, Whitechapel Road, E. Free. See p. 16. | Daily, 1.30 p.m. | .... |
| Middlesex Hospital, Berners Street, W. Free. See p. 16. | .... | Mr. Morris<br>Th., 1.30 |
| St. Saviour's Hospital, Osnaburg Street, N.W. By Governors' letters. | Dr. A. Kennedy | Daily, 10.30 and 4 |

### CHILDREN'S DISEASES.

| Hospitals and Terms of Admission. | Physicians. | Surgeons. |
|---|---|---|
| Alexandra Hosp. for Children, 18, Queen's Sq., W.C. No out-patients. For hip disease | .... | Mr. H. Marsh<br>„ A. Bowlby |
| Belgrave Hosp. for Children, 79, Gloucester Street, Pimlico. By Governors' letters | Dr. W. Hope<br>„ W. Ewart | M., Tu., Th., F., 9 a.m. |
| Cheyne Hospital for Sick and Incurable Children, 46, Cheyne Walk, Chelsea, S.W. In-patients only. | Dr. G. V. Poore | Mr. J. Macready<br>„ J. P. Bartlett |
| East London Hospital for Children and Dispensary for Women, Glamis Road, Shadwell, E. By Governors' letter. Urgent accident cases free | Dr. E. Smith<br>„ H. Donkin<br>„ H. Crocker<br>„ J. A. Coutts<br>„ D. Williams<br>M., Tu., W., Th., F., 1 to 1.30 (new cases); 3 to 3.30 (old cases) | Mr. A. Caesar<br>„ H. A. Reeves<br>„ R. W. Parker<br>Tu., F., 9 a.m. |
| Evelina Hospital for Sick Children, Southwark Bridge Road, S.E. Free, but Governors' letter takes precedence | Dr. N. Tirard<br>„ F. Wilcocks<br>Daily, 9 a.m. | Mr. C. Symonds<br>„ G. Makins<br>Daily, 9 a.m. |
| Great Northern Central Hospital. Free. See p. 16. | W., 2.30 p.m. | .... |
| Grosvenor Hospital for Women and Children, Vincent Sq., Westminster, S.W. By Subscriber's letter or payment | Dr. A. C. Smythe<br>„ R. Gibbons<br>„ Steavenson<br>M., Tu., Th., S., 2 to 3 p.m. | Mr. R. Parker<br>M., Tu., Th., S., 2 to 3 p.m. |
| Hospital for Sick Children, 49, Great Ormond Street, W.C. By Governors' letters. Other cases investigated. Patients under 12 | Dr. Robt. Lee<br>„ Abercrombie<br>„ Lubbock<br>„ A. Money<br>Daily, 8.30 to 10 | Mr. Morgan<br>„ Pitts<br>W., S., 8.30 to 10 a.m. |
| King's College Hospital. By Governor's letter. See p. 16 | Dr. T. C. Hayes<br>W., F., 1 | .... |
| North Eastern Hospital for Children, Hackney Road, E. By subscriber's free ticket; or by payment of 4*d.* on admission, and 3*d.* per week | Dr. W. Cayley<br>„ F. C. Turner<br>„ C. E. Semple<br>„ W. Pasteur<br>M., W., Th., S., 1.30 | Mr. Waren Tay<br>„ R. Godlee<br>M., 1.30; F., 9 a.m. |
| Paddington Green Children's Hospital, Paddington Green, W. Admission free | Dr. T. Brunton<br>„ L. Ogilvie<br>„ G. Laycock<br>„ S. Phillips<br>Daily, 9.15 | Mr. W. Cheyne<br>„ S. Boyd<br>Daily, 9 a.m. |
| Royal Hospital for Children and Women, Waterloo Bridge Road, S.E. Boys over 12 not admissible | Dr. Park<br>„ Dakin<br>„ Hadden<br>Daily, 12 to 2 | Mr. Day<br>W., 12 to 2 |
| St. Mary's Hospital, Paddington. Children over 2. See p. 16. | M., Th., 1.30 | .... |
| St. Thomas's Hospital, Westminster Bridge Road See p. 16. | Dr. Cory<br>W., S., 1.30 | .... |
| Samaritan Free Hospital for Women and Children, Lower Seymour Street, W.; Branch, 1, Dorset St., Manchester Sq., W. Governor's letter or card is necessary if the disease of the applicant is *not* peculiar to her sex | Dr. M. Prickett<br>„ A. Routh<br>„ W. Griffiths<br>Daily, 12 to 2 | Mr. W. Meredith<br>„ A. Doran<br>„ J. Malcolm<br>Daily, 12 to 2 |
| Victoria Hosp. for Children, Queen's Road, Chelsea, S.W. Out-patients admitted by letter, available for a fortnight. Age of boys, 2 to 12 years; age of girls, 2 to 16 | Dr. A. Venn<br>„ C. Fox<br>„ J. Drewitt<br>„ T. Philpot<br>M., 9, 1.30; Tu., W., 9; Th., 9, 1.30; F., 9, 1.30; S., 9.30 | Mr. F. Churchill<br>„ Walter Pye<br>M., 9.30; Tu., 10; Th., 9.30; F., 10 |

### CONSUMPTION & DISEASES OF THE CHEST.

| Hospitals and Terms of Admission. | Medical Officers, and Days and Hours of Attendance. Physicians. | Surgeons. |
|---|---|---|
| City of London Hospital for Diseases of the Chest, Victoria Park, E. By subscriber's letter available for 6 weeks. Examinations, S. | Dr. V. D. Harris<br>„ J. A. Ormerod<br>„ E. C. Beale<br>„ J. Anderson<br>„ B. Fenwick<br>„ Angel Money<br>„ L. E. Shaw | Daily, 2 p.m. |
| Hospital for Consumption, Brompton, S.W. By subscriber's letter | Dr. T. H. Green<br>„ J. M. Bruce<br>„ J. K. Fowler<br>„ P. Kidd<br>„ C. Y. Biss<br>„ T. D. Acland | Daily, 10 a.m. |
| Infirmary for Consumption, 26, Margaret St., Cavendish Sq., W. By subscriber's letter available for 8 weeks | Dr. Cooper Torry<br>„ Jagielaki<br>„ J. G. Barratt<br>Daily, 2 p.m. | Mr. F. Carr Beard<br>Daily, 2 p.m. |
| North London Hospital for Consumption, Mount Vernon, Hampstead, N. Admission by subscriber's letter available for 6 weeks | Dr. A. Evershed<br>„ E. Haward<br>„ J. Shaw<br>„ B. O'Connor<br>„ G. D. Pidcock<br>„ S. Taylor<br>„ J. L. Cassidy<br>„ J. E. Squire<br>„ W. Boulting<br>M., Tu., W. F., 10.30. | Out-patients also seen daily, 1 p.m., at branch 216, Tottenham Court Road. |
| Royal Hospital for Diseases of the Chest, City Road, E.C. By Governor's letter | Dr. Hensley<br>„ Gilbert Smith<br>„ Finlay<br>„ Murrell<br>„ White<br>„ Pringle<br>M., Tu., W., Th., F., 2; S., 9 a.m. | Mr. A. P. Gould<br>M., Tu., W., Th., F., 2; S., 9 a.m. |
| Royal National Hospital for Consumption and Diseases of the Chest, Ventnor. The London offices are at 34, Craven St., Strand. Admit only incipient cases | Dr. J. Cockle<br>„ H. Weber<br>„ H. Port<br>„ Thorowgood<br>„ A. Davis<br>„ J. G. S. Coghill<br>„ R. Robertson | Mr. J. Whitehead<br>„ Williamson<br>Out-patients seen daily at Ventnor, 11 a.m. |

### EAR CASES.

| Hospitals and Terms of Admission. | Physicians. | Surgeons. |
|---|---|---|
| Central London Throat and Ear Hospital, Gray's Inn Road, W.C. Free, but when able, a small payment is expected | M., W., Th., S., 2; Tu., F., 5 p.m. | Mr. J. D. Grant<br>„ P. S. Jakins<br>„ T. W. C. Jones |
| Great Northern Central Hospital. See p. 16. | W., 9 | Mr. A. E. Cumberbatch |
| Guy's Hospital, St. Thomas's St., S.E. See p. 16. | Tu., F., 12.30 | Mr. Laidlaw-Purves |
| King's College Hospital, Portugal Street, W.C. See p. 16. | Th., 1 | Mr. U. Pritchard |
| London Hospital, Whitechapel Road, E. See p. 16. | S., 9 | Mr. E. Woakes<br>„ T. M. Hovell |
| London Homœopathic Hospital. By registration card at Hospital | Dr. Cooper | S., 3 p.m. |
| Metropolitan Ear and Throat Infirmary, 13, Howland St., W. By subscribers' letters, or by payment; free to very poor | Dr. Pickett<br>„ D. Nesbitt | Daily, 2.30; W., 7 p.m. |
| Middlesex Hospital, Berners Street, W. See p. 16. | Tu., 9 a.m. | Mr. Hensman |
| Municipal Throat and Ear Infirmary, 266, City Road, E.C. Free to necessitous; or by payment | Dr. G. Holmes<br>„ J. A. Hatch | M., W., F., 10 to 12; Tu., Th., 6 to 8 p.m. |
| Royal Ear Hospital, Frith Street, Soho, W. Admission by subscriber's ticket; free to the very poor | Dr. U. Pritchard<br>„ Farquhar Matheson | Tu., F., 9 to 12; M., S., 3 to 5 |
| St. Bartholomew's Hospital, Smithfield, E.C. See p. 16. | Tu., F., 2 | Mr. Cumberbatch |
| St. George's Hospital, Hyde Park, W. See p. 16. | Tu., 2 | Mr. Dallery |
| St. Mary's Hospital, Paddington. By Governors' letters. See p. 16 | W., S., 9.30 | Mr. G. P. Field |
| St. Thomas's Hospital, Westminster Bdg. Rd., S.E. See p. 16. | M., 1.30 | Mr. Clutton |
| University College Hospital. By Governors' letters. See p. 16 | S., 9 a.m. | Mr. Barker |
| Westminster Hospital. By Governors' letters. See p. 16 | .... | Tu., F., 9 |

### EYE DISEASES.

| Hospitals and Terms of Admission. | Medical Officers, and Days and Hours of Attendance. Physicians. | Surgeons. |
|---|---|---|
| Central London Ophthalmic Hospital, Gray's Inn Rd., W.C. Admission free | Daily, 1 | Mr. T. B. Archer, „ G. Abbott, „ W. Charnley, „ W. H. Jessop, „ E. Clarke, „ J. R. Kemp |
| Great Northern Central Hospital, Caledonian Rd., N. Free | Tu., F., 9.30 | Mr. Hutchinson |
| Guy's Hospital, St. Thomas's Street, S.E. See p. 16 | Tu., F., 12.30 | Mr. Higgins, „ Brailey |
| Hospital for Sick Children, 49, Gt. Ormond Street, W.C. | Tu., 2 | Mr. R. M. Gunn |
| King's College Hospital. By Governors' letters. See p. 16 | M., F., 1 | Mr. McHardy |
| London Hospital. By Governors' letters. See p. 16 | Tu., S., 9 | Mr. Eve, „ Waren Tay |
| London Homœopathic Hospital, Great Ormond Street, W.C. | Dr. Byres Moir | M., Th., 3 |
| Middlesex Hospital. By Governors' letter. See p. 16 | Tu., F., 9 | Mr. Lang |
| North-West London Hospital. By Governors' letters. See p. 16 | F., 9.30 | Mr. J. Collins |
| Paddington Green Children's Hospital. Free. See p. 32 | Th., 9 | Mr. W. H. Jessop |
| Royal Free Hospital, Gray's Inn Road, W.C. | M., F., 9 | Mr. Mackinley |
| Royal London Ophthalmic Hospital, Blomfield Street, Moorfields, E.C. Admission free | Daily, 8 | Mr. Streatfield, „ J. W. Hulke, „ G. Lawson, „ J. Cowper, „ W. Tay, „ J. Tweedy, „ E. Nettleship, „ Gunn, „ Lang |
| Royal South London Ophthalmic Hospital, 6, St. George's Circus, Southwark, S.E. Free to the necessitous poor; or by payment | Daily, 2 | Mr. Watson, „ McHardy |
| Royal Westminster Ophthalmic Hospital, King William Street, W.C. Free to accidents and the indigent poor; or by payment | Daily, 12 to 1.30 | Mr. Power, „ Rouse, „ Cowell, „ Macnamara, „ Juler, „ Frost, „ Hartridge |
| St. Bartholomew's Hospital, Smithfield, E.C. See p. 16 | Tu., Th., 2; S., 2.30 | Mr. Power, „ Vernon |
| St. George's Hospital. By Governors' letters. See p. 16 | W., S., 2 | Mr. Frost |
| St. Mary's Hospital. By Governors' letters. See p. 16 | Tu., F., 9.30 | Mr. H. E. Juler |
| St. Thomas's Hospital, Westminster Bdg. Rd., S.E. See p. 16 | Tu., Th., F., 1.30 | Mr. Nettleship, „ Lawford |
| University College Hospital. By Governors' letters. See p. 16 | M., Tu., Th., F., 2 | Mr. J. Tweedy |
| West London Hospital. By Governors' letters. See p. 16 | Tu., Th., S., 2 | Mr. Vernon, „ Dunn, M., Th., 2 |
| Westminster Hospital. By Governors' letters. See p. 16 | .... | |
| Western Ophthalmic Hospital, 153, Marylebone Road, W. By small payments | Dr. Miller, „ Collins, „ Wheatley, M., Th., F., 2 | Mr. Buxton, „ Barrett, „ C. Jones, Tu., W., F., S., 2 |

### HEART, PARALYSIS, EPILEPSY, AND DISEASES OF THE NERVOUS SYSTEM GENERALLY.

| Hospitals and Terms of Admission. | Physicians. | Surgeons. |
|---|---|---|
| Hospital for Epilepsy and Paralysis, etc., 32, Portland Terrace, Regent's Park Road, N.W. By payment, but free to the necessitous poor | Dr. J. Althaus, „ A. H. Bennett, „ G. Ogilvie, „ G. L. Laycock, M., Tu., W. Th., F., 2 | Mr. J. A. Bloxam, „ A. P. Gould, M., Tu., W., Th., F., 2 |
| National Hospital for Diseases of the Heart, 32, Soho Sq., W. By Governors' letters, but to poor without letter | Dr. V. Ambler, „ A. Duncan, „ R. Vorley, „ J. Murray, M., 2, 7; Tu., 3; W., 2, 8; Th., 2; F., 3 | Mr. G. Bennett, Tu., 5 |
| National Hospital for the Paralysed and Epileptic, Queen Square, W.C. By subscriber's letter | Dr. H. C. Bastian, „ W. R. Gowers, „ D. Ferrier, M., Tu., W., F., 1.30 | Mr. W. Adams, „ V. Horsley, M., Tu., W., F., 1.30 |
| West End Hospital for Diseases of the Nervous System, 73, Welbeck Street, W. By subscriber's letter | Dr. Tibbitts, „ Stretch-Dowse, „ S. H. Armitage | M., Tu., W., Th., 1.3c; F., 6 |

### ORTHOPÆDIC CASES AND DEFORMITIES OF BODY GENERALLY.

| Hospitals and Terms of Admission. | Medical Officers, and Days and Hours of Attendance. Physicians. | Surgeons. |
|---|---|---|
| City Orthopædic Hospital, 27, Hatton Garden, E.C. Free | Tu., F., 2.0. | Mr. E. J. Chance, „ J. Addison |
| National Orthopædic Hospital, 234, Great Portland Street, W. Free | M., Tu., Th., F., 2.0; W., 1 | Mr. F. R. Fisher, „ W. J. Roeckel, „ E. M. Little |
| Royal Orthopædic Hospital, 297, Oxford Street, W. By Governor's letter | Daily, 1.0 | Mr. Broadhurst, „ H. A. Reeves, „ C. Read, „ W. Balkwill, „ H. F. Baker |
| St. Bartholomew's Hospital, Smithfield, E.C. Free. See p. 16 | M., 2.30 | Mr. Walsham |
| St. George's Hospital, Hyde Park, S.W. See p. 16 | W., 2.0 | Mr. Bennett |

### SKIN, DISEASES OF.

| Hospitals and Terms of Admission. | Physicians. | Surgeons. |
|---|---|---|
| British Hospital for Diseases of the Skin (West Branch), 61, Great Marlborough St., W. By payment; necessitous free | M., 2; W., 7; F., 2 | Mr. B. Squire, „ G. Gaskoin |
| ———South London Branch, 5, Newington Butts, S.E. | M., 7; W., 2; F., 7 | Ditto |
| Charing Cross Hospital. By Governor's letter. See p. 16 | Dr. Sangster | M., 1.30 |
| Guy's Hospital. By payment of 3*d.* each | Dr. Pye-Smith, „ Hale-White | Tu., 12.30 |
| Hospital for Diseases of the Skin, 52, Stamford Street, S.E. Free and by payment | M., W., 3; Tu., Th., F., 2 | Mr. Waren Tay, „ W. Cottle |
| King's College Hospital. By Governor's letter. See p. 16 | .... F. 1 | .... |
| London Hospital. By Governor's letter. See p. 16 | Dr. Mackenzie | Th., 9 |
| London Homœopathic Hospital. By registration card. | Dr. G. Blackley | Th., 3 |
| Middlesex Hospital. By Governor's letter. See p. 16 | Dr. R. Liveing | F., 4 |
| National Institution for Diseases of the Skin, 227, Gray's Inn Road, W.C. By payment, or free on certificate of a medical man or minister | Dr. B. Meadows | M., Th., 6 |
| North-West London Hospital. By Governor's letter. See p. 16 | Dr. J. Stowers | Tu., 1.30 |
| Paddington Green Children's Hospital. Free. See p. 32 | Dr. T. C. Fox | S., 9.15 |
| St. Bartholomew's Hospital. Free. See p. 16 | F., 1.30 | Mr. Cripps |
| St. George's Hospital. By Governor's letter. See p. 16 | Dr. Cavafy | W., 2 |
| St. John's Hospital for Skin Diseases, Leicester Sq., W.C. By Governor's letter or payment | Dr. Robinson, „ Dow, „ Harries, M., W., Th., 2; M., W., F., 7 | Mr. J. Milton, M., Tu., Th., F., 2 |
| ———Markham Square, Chelsea (Branch) | Dr. Harries, „ Campbell | M., Th., 10 |
| St. Mary's Hospital. By Governor's letter. See p. 16 | M., Th., 9.30 | Mr. M. Morris |
| St. Thomas's Hospital. By letter. See p. 16 | Dr. Payne | W., 1.30 |
| University College Hospital. By Governor's letter. See p. 16 | Dr. R. Crocker | W., 1.45; S., 9.15 a.m. |
| Westminster Hospital. By Governor's letter. See p. 16 | .... W., 1.30 | .... |

### STONE AND OTHER DISEASES OF URINARY ORGANS.

| Hospitals and Terms of Admission. | Physicians. | Surgeons. |
|---|---|---|
| St. Peter's Hospital for Stone, Henrietta Street, W.C. Admission free | *Men*, M., Tu., Th., 2 to 3; W., 5 to 7; S., 4 to 7; *Women and children*, F., 2 to 3 | Mr. E. Fenwick, „ F. Edwards, „ Heycock |

### DISEASES & IRREGULARITIES OF THE TEETH.

| Hospitals and Terms of Admission. | Physicians. | Surgeons. |
|---|---|---|
| Charing Cross Hospital. By Governor's letter. See p. 16 | M., W., F., 9.0 | Mr. J. Fairbank |
| Dental Hospital of London, Leicester Sq., W.C. Free to the poor, together with any operative assistance that may be immediately necessary. For special operation by Governor's letter | Daily, 9 to 11 | Mr. D. Hepburn, „ Woodhouse, „ G. Gregson, „ S. Bennett, „ F. Canton, „ H. Moor, „ W. Hern, „ Underwood, „ C. Rogers, „ Parkinson, „ L. Read, „ C. E. Trumen |

### DISEASES, Etc., OF THE TEETH.—*Continued.*

| Hospitals and Terms of Admission. | Medical Officers, and Days and Hours of Attendance. Physicians. | Surgeons. |
|---|---|---|
| German Hospital. Free to Germans. See p. 16 | Th., 10 to 11 | Mr. A. P. Reboul; „ C. West |
| Great Northern Central Hospital. Free. See p. 16 | W., 1.30 | Mr. Keen |
| Guy's Hospital. On payment of 3*d*. See p. 16 | Tu., Th., F., 12.30 | Mr. Moon; „ Newland; „ Pedley |
| Hospital for Consumption, Brompton. By Governor's letter. | W., 2.0 | Mr. C. J. Noble |
| Hospital for Sick Children, Gt. Ormond Street, W.C. | W., 9.0 | Mr. Cartwright |
| King's College Hospital. By Governor's letter. See p. 16 | Tu., F., 10.0 | Mr. Cartwright |
| London Hospital. By Governor's letter. See p. 16 | Tu., 9.0 | Mr. A. Barrett |
| London Homœopathic Hospital. By registration card. See p. 16 | M., 9.0 | Mr. Cronin |
| Metropolitan Free Hospital. Free See p. 16 | Tu., Th., S., 9.0 | Mr. G. Curnock |
| Middlesex Hospital. By Governor's letter. See p. 16 | M., F., 9.30 | Mr. Bennett; „ Hern |
| National Dental Hospital, 149, Gt. Portland Street, W. By Governor's letter | Daily, 9 to 11 | Mr. Williams; „ H. Rose; „ A. F. Canton; „ F. H. Weiss; „ T. Gaddes; „ A. Smith; „ W. G. Weiss; „ W. Humby; „ G. Bradshaw; „ M. Davis; „ H. G. Read; „ W. Thomson |
| National Hospital for Disease of Heart. By Governor's letter. | W., 11.0 | Mr. J. Fitzgerald |
| North-West London Hospital. By Governor's letter. | F., 9.0 | Mr. W. Maggs |
| Paddington Green Children's Hospital. Free. See p. 32 | W., 9.0 | Mr. C. Cotterell |
| Royal Hospital for Children. By Governor's letter. | F., 1.30 | Mr. Whitehouse |
| St. Bartholomew's Hospital. Free. See p. 16 | Tu., F., 9.0 | Mr. Ewbank; „ Paterson |
| St. George's Hospital. By Governor's letter. See p. 16 | Tu., S., 9.0 and 1.0 | Mr. Winterbottom |
| St. Mary's Hospital. By Governor's letter. See p. 16 | W., S., 9.30 | Mr. H. Hayward |
| St. Thomas's Hospital. Free. See p. 16 | Tu., F., 10.0 | Mr. Truman |
| University College Hospital. By Governor's letter. See p. 16 | W., 9.30 | Mr. Hutchinson |
| Victoria Hospital for Children. See p. 32 | S., 9.0 | Mr. Francis Fox |
| West London Hospital. By Governor's letter. See p. 16 | Tu., F., 9.30 | Mr. Albert |
| Westminster Hospital. By Governor's letter. See p. 16 | W., 9.15 | Mr. J. Walker; „ M. Smale |

### THROAT DISEASES.

| Hospitals and Terms of Admission. | Physicians. | Surgeons. |
|---|---|---|
| Central London Throat and Ear Hospital. See p. 32 | .... | M., W. Th., S., 2; Tu., F., 5 |
| Great Northern Central Hospital. Free. See p. 16 | W., 2 | Mr. Watson |
| Guy's Hospital. On payment of 3*d*. | Th., 1 | Mr. Symonds |
| Hospital for Diseases of the Throat. By payment, but free to the necessitous poor | Daily, 1.30; Tu., Th., 6.30 p.m. | Mr. E. Woakes; „ T. Hovell; „ G. Stoker; „ Fenton-Jones |
| Infirmary for Consumption 26, Margaret Street, W. See p. 16 | .... | Daily, 2 |
| King's College Hospital. By Governor's letter. See p. 16 | Tu. 1 | .... |
| Metropolitan Ear and Throat Infirmary. See p. 32 | Daily, 2.30; W., 7 p.m. | .... |
| Middlesex Hospital. By Governor's letter. See p. 16 | Tu., 9. | Mr. Hensman |
| Municipal Throat and Ear Infirmary. See p. 32 | Dr. Gordon-Holmes; „ J. A. Hatch | M., W., F., 10 to 12; Tu., Th., 6 to 8 p.m. |
| North West London Hospital. By Governor's letter. See p. 16 | M., 3 | .... |
| St. Bartholomew's Hospital. Free. See p. 16 | F., 2.30 | Mr. Butlin |
| St. George's Hospital. By Governor's letter. See p. 16 | Th., 2 | Mr. Whipham |

### THROAT DISEASES.—*Continued.*

| Hospitals and Terms of Admission. | Medical Officers, and Days and Hours of Attendance. Physicians. | Surgeons. |
|---|---|---|
| St. Mary's Hospital. By Governor's letter. See p. 16 | M., Th., 9.30 a.m | Mr. A. Norton |
| St. Thomas's Hospital. Free. See p. 16 | Dr. Semon | Tu., F., 1.30 |
| University College Hospital. By Governor's letter. See p. 16 | Dr. Poore | Th., 1.30 |
| Westminster Hospital. By Governor's letter. See p. 16 | W., 9 | .... |

### DISEASES OF WOMEN (OBSTETRIC CASES, ETC.).

| | | |
|---|---|---|
| Charing Cross Hospital. By Governor's letter. See p. 16 | Dr. Amand Routh | Tu., 1.30 |
| Chelsea Hospital for Women, Queen's Elm, Fulham Road. For reception of gentlewomen in reduced circumstances, and respectable poor women. Payment from 10*s*. 6*d*. a week. Gratuitous treatment to poor on recommendation of a Governor | Dr. V. Dickinson; „ J. Mackern; „ W. Travers; „ G. Harper; „ Fenton-Jones; „ J. T. Parsons; „ J. Phillips; „ H Rutherford | M., Tu., W., F., 2. New patients, 1.30 |
| East London Hospital for Children and Infirmary for Women. See p. 32 | M., Tu., W., Th., F., 1 to 1.30 (new cases); 3 to 3.30 (old cases) | .... |
| Great Northern Central Hospital. Free. See p. 16. | Dr. F. Barnes | W., 2 |
| Grosvenor Hospital for Women and Children. See p. 32 | M., Tu., Th., S., 2 to 3 | .... |
| Guy's Hospital. Free without letter (Uterine Wards) See p. 16 | Dr. Horrocks | Th., S., 12.30 |
| Hospital of St. John and St. Elizabeth, 50, Great Ormond Street, W.C. For females suffering from advanced or long-standing disease. No out-patients. | Dr. Constable; „ Tegart | Patients examined on Th. at 3 |
| Hospital for Women, Soho Sq., W. Free to out-patients. | Dr. M. Moullin; „ B. Fenwick; „ Holland; „ R. T. Smith; „ Oliver; M., 11; Tu., W., F., S., 10; Th., 11, 2. | Mr. S. Osborn; Th., 2 |
| King's College Hospital. By Governor's letter. See p. 16 | Dr. T. C. Hayes | W., F., 1 |
| London Homœopathic Hospital. By registration card. See p. 16 | Dr. Carfral | Tu., 3 |
| Metropolitan Free Hospital. Free. See p. 16 | Dr. A. Venn | W., 2 |
| Middlesex Hospital. By Governor's letter. See p. 16 | Dr. W. Duncan | W., S., 1.30 |
| New Hospital for Women, 222, Marylebone Road. By subscriber's letter. Out-patients pay 6*d*. entrance fee, and 2*d*. each visit. All the physicians are women. | Dr. G. Anderson; „ Watkins; „ Marshall; „ De la Cherois; M., Tu., W., Th., F., 1 to 3; Sat., 9 to 10 a.m. | Mr. G. Lawson; „ A. Norton; „ T. Smith; „ W. Meredith; M., T., W., Th., F., 1 to 3; S., 9 to 10 a.m. |
| North-West London Hospital. By Governor's letter, or payment | W., 1.30 | .... |
| Royal Free Hospital, Gray's Inn Road. Free. See p. 16 | Dr. Hage | S., 2 |
| Royal Hospital for Children and Women. By Governor's letter and payment of 1*d*. per week | Dr. Park; „ Dakin; „ Hadden; „ Duncan; Daily, 12 to 2 | Mr. Day; W., 12 to 2 |
| St. Bartholomew's Hospital. Free. See p 16 | Dr. Gadson | W., S., 9 |
| St. George's Hospital. By Governor's letter. See p. 16 | Dr. Champeneys | M., Th., 2 |
| St. Mary's Hospital. By Governor's letter See p. 16 | Dr. A. Meadows; „ Handfield-Jones | M., Th., 1.30 |
| St. Thomas's Hospital. Free. See p. 16 | Dr. Cory | W., S., 1.30 |
| Samaritan Free Hospital for Women and Children. See p. 32 | Daily, 12 to 2 | .... |
| University College Hospital. By Governor's letter. See p. 16 | Dr. Williams | Tu., F., 2 |
| West London Hospital. By Governor's letter. See p. 16 | Dr. M. Moullin | M., S., 2 |
| Westminster Hospital. By Governor's letter. See p. 16 | Dr. Potter; „ Grigg | Tu., F., 9 |

☞ Secretaries will oblige by communicating necessary corrections from time to time.

